# Detection of degenerate Stokes index states

**DOI:** 10.1038/s41598-020-77365-8

**Published:** 2020-11-27

**Authors:** Gauri Arora, S. Deepa, Saba N. Khan, P. Senthilkumaran

**Affiliations:** Department of Physics, Indian Institute of Technology Delhi, Hauz Khas, New Delhi 110016 India

**Keywords:** Optics and photonics, Physics

## Abstract

Stokes phase is the phase difference between orthogonal component states in the decomposition of any polarization state. Phase singularities in the Stokes phase distribution are Stokes singularities of an inhomogeneous polarization distribution. Under circular decomposition, Stokes phase distribution $(\phi _{12})$ represents polarization azimuth $(\gamma )$ distribution and the singularities present in it are polarization singularities. Therefore, the charge of the Stokes vortices depicted as Stokes index $\sigma _{12}$ is an important parameter associated with the polarization singularity. The Hybrid order Poincaré sphere (HyOPS)/Higher order Poincaré sphere (HOPS) beams, all having same Stokes index, contain a Stokes singularity at the center of the beam as these beams are constructed by vortex superposition. These beams, being superposition of orthogonal orbital angular momentum (OAM) states in orthogonal spin angular momentum (SAM) states can offer great multiplexing capabilities in communication. In this article, we identify these degenerate Stokes index states and discuss the ways and means of lifting this degeneracy. Otherwise, there are limitations on intensity based detection techniques, where demultiplexing or segregation of different HOPS/HyOPS beams is warranted. The method adduced here uses the diffraction of these beams through an equilateral triangular aperture in combination with polarization transformation as a probe to lift the Stokes index/Stokes phase degeneracy. Successively, the novelty of the detection scheme is discussed in the context of beams with alike polarization distributions where even the technique of Stokes polarimetry fails to predict the OAM and SAM content of the beam.

## Introduction

Increased interest in the use of spin-orbit beams invites simple detection methods. Phase singular beams carrying orbital angular momentum (OAM) have immense applications^1,2^ and the techniques based on diffraction and interference are known to unambiguously detect the topological charge of such scalar fields. Spin-orbit beams are beams embedded with polarization singularities^3–6^ and can be described as a superposition of orthogonal OAM states in orthogonal spin angular momentum (SAM) states. In recent years, beams with single polarization singularity at the center of the beam are called by special names such as Poincaré beams^7^, cylindrical vector beams^8^, vector vortex beams^9^, Majorana beams^10^. Here, we deal with higher order Poincaré sphere (HOPS) beams^11–13^ and hybrid order Poincaré sphere beams (HyOPS)^14–17^ that contain a polarization singularity at the center of the beam and only two dimensional field distributions are considered. In recent years, many methods have been reported to generate HyOPS^15,16,18,19^ and HOPS beams^20–24^. It is also possible to have multiple polarization singularities in a single beam like in random fields^25,26^ and lattice fields^27,28^. Stokes phase is more fundamental to deal singularities appearing in polarized light fields. In this article, we discuss the degeneracy in Stokes index, that is used to characterize the Stokes phase distribution. Although the discussion presented here is valid for Stokes singularity with any index, the degeneracies are explained by choosing a particular index.

The endowed polarization singularities are broadly classified as ellipse and vector fields singularities. Ellipse field singularities are isolated points or lines where some property of elliptical state of polarization is indeterminate whereas vector field singularities are point singularities where the orientation of the electric field vector is not defined^4,29,30^. The predominant state of polarizations (SOPs) around an ellipse field and vector field singularities are ellipses and linear states respectively. C-points and L lines are two types of ellipse field singularities whereas V-point is vector field singularity. C-points are points of circular polarization in an inhomogeneously polarized ellipse field where azimuth of polarization is indeterminate and L line is a continuous line of linear polarization states in ellipse field where handedness is not defined. Since HOPS beams are constant ellipticity fields, they are useful to represent V-points. In HyOPS beams, both ellipticity and azimuth are variables and are useful to represent C-points.

Polarization of light can be described using four measurable Stokes parameters $S_0$, $S_1$, $S_2$ and $S_3$^31,32^. For inhomogeneously polarized beams, complex field distribution $S_{jk}(x,y) = S_j(x,y) +iS_k(x,y)$ constructed using Stokes parameters, provides a convenient way to study Stokes singularities. Here, indices $\{j,k\}$ run cyclically from {1,2,3} where $j \ne k$. Stokes phase is $\phi _{jk}=Arg(S_j+iS_k)$. Phase singularities present in Stokes phases are called Stokes singularities^33^. The polarization singularities which are phase singularities in $\phi _{12}$ Stokes phase are subset of Stokes singularities. C-point and V-point polarization singularities are points in inhomogeneous polarization distribution in which the azimuth ($\gamma$) is indeterminate^5,27,34–36^. This azimuth distribution is related to $\phi _{12}$ Stokes phase distribution as $2\gamma (x,y) =\phi _{12}(x,y)$. Around a polarization singular point, a circulating Stokes phase gradient is present. The Stokes index is defined as $\sigma _{jk} = (\Delta \phi _{jk})/(2\pi )$ where $\Delta \phi _{jk}$ is the total accumulated phase around the singular point^34^.

Phase singular beams with unequal topological charges are orthogonal. These are beams embedded with scalar field singularities. The orbital angular momentum content in these beams can be found by the distinct diffraction patterns they produce^37–42^. But for polarization singular beams, the diffraction patterns alone will not suffice^43–48^ for their unambiguous discrimination. The signature diffraction patterns of singular beams are due to the disintegration of higher charge singularity into multiple lower ones. In scalar singularities, the phase vortex of higher charge disintegrates into multiple generic lower charge singularities, where topological charge conservation is observed. For polarization singularities, owing to multiple parameters associated with polarization of light, the disintegrated fragment singularities have multiple attributes. The V-points and higher-order C-points transform into lower-order more stable elliptic field singularities under small perturbation following index and sign conservation rules^25,26,30,34,49^. Reports on V-point diffraction show that there is index and helicity conservation^44,46,47^.

**Figure 1:**
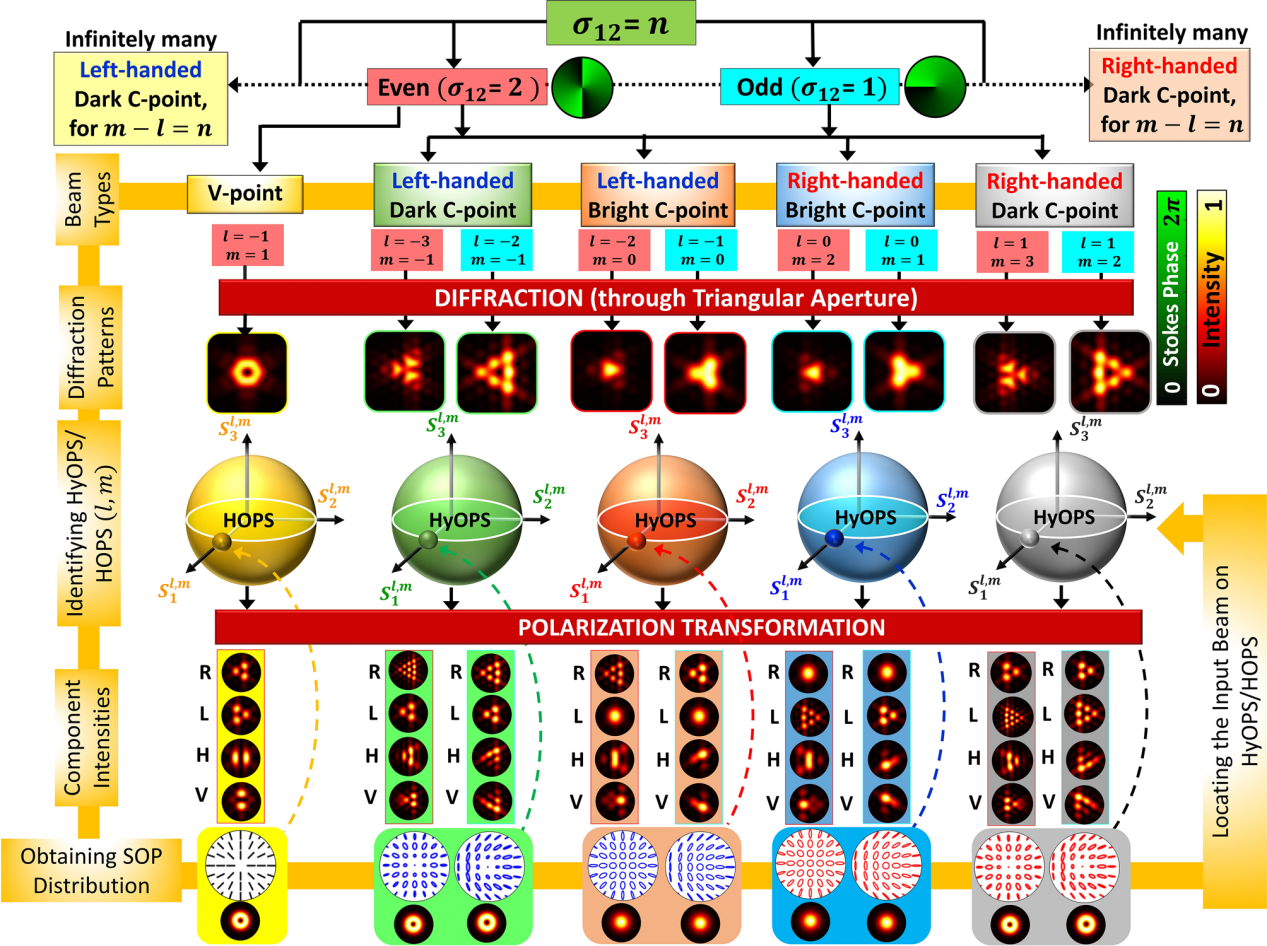
Flow chart summarizing the detection scheme of degenerate Stokes index states.

Ellipse field singularities have C-point index defined as $I_c=\frac{1}{ 2\pi } \oint \nabla \gamma \cdot dl$ whereas vector field singularities have V-point index also called as Poincare-Hopf index defined as $\eta = \frac{1}{ 2\pi } \oint \nabla \gamma \cdot dl$. Here, *dl* is the closed path of integration around the singular point. Both the indices are defined by using the azimuth $\gamma$ of the SOP. These singularities therefore have a common Stokes index $\sigma _{12}= 2I_c = 2\eta$^33,34^. Stokes phase distribution $(\phi _{12})$ has often been used to identify the presence of polarization singularities in an inhomogeneous field. Since the information about the Stokes parameter $S_3$ is absent in Stokes phase construction ($\phi _{12}$), determination of handedness of ellipse field singularities and distinction between vector and ellipse field singularities are not possible. Further, the Stokes phase $\phi _{12}=\phi _L-\phi _R$, is the phase difference between left and right circular polarization components of a given SOP distribution. Suffixes R and L stand for right and left circular polarization component states respectively. Therefore many spin-orbit beams (HOPS/HyOPS beams) share a common Stokes index and Stokes phase distribution and are degenerate. It can be often construed wrongly by many that the index decides the SAM and OAM content in a beam. But actually for a given SOP distribution (given index), there can be various combinations of SAM and OAM states possible. This comes out of the phenomenon of degeneracy in the index.

The only method that can detect/lift the Stokes phase/Stokes index degeneracy present in such inhomogeneous beams is Stokes polarimetry. But that has also not been discussed in the context of degenerate Stokes index states anywhere in the existing literature. Moreover, there are cases wherein the beams composed of different OAM state combination (or different relative weightage of OAM states) possess alike polarization distribution where even Stokes polarimetry fails to distinguish between such beams. For example, a dark C-point of given Stokes index (say $\sigma _{12}= 2$) and polarization distribution (say radial C-point beams) can be generated using many combinations of optical vortices ((1,3),(2,4),(3,5)...). Each of these HyOPS beams are associated with different OAM content. Also, the corresponding Gouy phase^50^ governed by the mode indices of the component states is different for the respective HyOPS beams. The propagation properties of these beams are entirely different from each other and the distinction between such dark C-point beams is needed to deploy it for a specific application. Notably, partially coherent dark C-point beams carrying different OAM content are reported to show different strength of anti-depolarization effect although the SOP distribution is alike^51^. Another case where one can encounter alike polarization distribution for two different beams is when the relative weightage of the two OAM states comprising the HyOPS beam is different. In both the above cases, if HyOPS beams are diffracted by an appropriately chosen aperture the distinction in term of OAM content and the relative weightage of OAM states can be made. Following polarization transformation one can further accurately predict the Stokes phase and SOP distribution of the HyOPS beams as well. To the best of our knowledge, we are the first to present a novel technique based on diffraction and polarization transformation to detect and efficiently distinguish these degenerate Stokes index states. These polarization structured spin-orbit beams find potential application in optical activity measurement^52^, achieving isotropic and directional edge enhancement^5^, designing robust beams for propagation in turbulent medium^53^, optical trapping and micro-manipulation^54^, Mueller matrix polarimetry^55^, and polarization speckle generation^56^.

In this work, we present the degenerate $\sigma _{12}$ Stokes index states and discuss the ways and means of lifting this degeneracy. A two-step process based on diffraction and polarization transformations is adduced here. The key advantage of the presented scheme is that one can even lift the degenerate Stokes phase states possessing alike SOP distribution (polarization distribution degeneracy) and can quantify the OAM and SAM content associated with the beam. The method allows the use of intensity detectors in demultiplexing or segregation of different HOPS/HyOPS beams. This article is organized as follows. In section “Methods”, we first introduce what forms degenerate set of spheres corresponding to a particular Stokes index/phase. This is followed by a detailing of our technique used to remove this degeneracy. In the next subsection, our experimental setup for detecting these degenerate Stokes index states is described. In Section “Results and Discussion”, all types of degeneracies that include Stokes index, Stokes phase, polarization distribution and diffraction pattern degeneracy that can exist in various HOPS/HyOPS beams are detailed. The experimental and theoretical diffracted patterns for the beam and its component states are shown to accurately reveal the component’s OAM and SAM states of the HOPS/HyOPS beam. The impact of our technique is accentuated by distinguishing the beams carrying alike polarization distribution but different OAM content/relative weight of OAM states. Finally, in the last section, the key findings of the research are concluded.

## Methods

### Degenerate spheres

Polarization singular beam as superposition of OAM and SAM states can be expressed as^5^:

1 $$\begin{aligned} {\left| {\psi _{l,m}(r,\theta )}\right\rangle }=\psi _{R}^l(r)\left| {R}_l\right\rangle +\psi _{L}^m(r)\left| {L}_m\right\rangle \end{aligned}$$

where

2 $$\begin{aligned} \left| {R}_l\right\rangle= & {} \frac{1}{\sqrt{2}} \exp (il\theta )(\hat{x}+i\hat{y}) \end{aligned}$$

3 $$\begin{aligned} \left| {L}_m\right\rangle= & {} \frac{1}{\sqrt{2}} \exp (im\theta )(\hat{x}-i\hat{y}) \end{aligned}$$

$\left| {R}_l\right\rangle$ and $\left| {L}_m\right\rangle$ represent right and left circularly polarized vortex beams with topological charges *l* and *m* respectively. $\psi _{R}^l$ and $\psi _{L}^m$ are their complex coefficients. ‘r’ and ‘$\theta$’ are radial and azimuthal coordinates expressed as $r=\sqrt{x^2+y^2}$ and $\theta =\tan ^{-1}(\frac{y}{x})$. All the possible polarization distributions that can be generated using Eq. (1) can be mapped on to a sphere in which the states $\left| {R}_l\right\rangle$ and $\left| {L}_m\right\rangle$ are represented by polar points. Each of the superposition state can be represented by a point on the surface of the sphere. In this new construct, a sphere with $l=-m$ is called higher order Poincaré sphere and a sphere with $l\ne m$ is called hybrid order Poincaré sphere^11–16^. For each combination of *l* and *m*, a unique sphere can be constructed. For the polarization singularity described by Eq. (1), the Stokes phase is given by $\phi _{12}=(m-l)\theta+\theta_0$ (where $\theta_0$ is constant relative phase). For a given Stokes phase, there can be many polarization singularities, because *m* and *l* can assume any value as long as the value of $(m-l)$ remains constant. The superposition leads to C-point polarization singularity of index $I_c=(m-l)/2$ when $|m|\ne |l|$ and V-points when $m= -l$^57^. Bright C-points result when $l\ne m$ and either *l* or *m* is zero whereas dark C-points result when $l\ne m$ and both *l* and *m* are non zero.

For a given Stokes index $\sigma _{12}$ there can be a multitude of SOP distributions represented by non-polar points on a HOPS or on a HyOPS. Further for a given Stokes index $\sigma _{12}$ (even) there can be one HOPS and many HyOPS possible as depicted in Fig. 1. For odd Stokes index beams, only HyOPSs are possible. The flow chart in Fig. 1 summarizes the detection scheme presented in the article by considering two cases of Stokes indices: odd ($\sigma _{12}=1$) and even ($\sigma _{12}=2$). All the SOP distributions that can be represented on a HOPS or a HyOPS (except polar points) and by all the HOPS and HyOPS with the same $\sigma _{12}$ index are degenerate Stokes index states. The points on a particular longitude of each sphere share the same Stokes phase distribution. Here, one Stokes phase state from each HyOPS and HOPS are selected in both cases to discuss the detection scheme. In the following sections, we choose a particular Stokes index value (|$\sigma _{12}|=2$) to explain the possible degeneracies that can occur. Similar analysis can be carried out for any arbitrary value of Stokes index.

### Removal of degeneracy

The two-step process adduced here for lifting the degeneracy in the Stokes index states is (i) diffraction of polarization singularities through equilateral triangular aperture and (ii) polarization transformation. Five representative degenerate Stokes phase states for a given Stokes index $\sigma _{12}$ are chosen to describe the diffraction and polarization transformation patterns. Four singularities are from four different HyOPS and one is from a HOPS (see Fig. 1). These are right and left-handed bright C-points, right and left-handed dark C-points, and V-point polarization singularities. These chosen singularities are often encountered and are represented by the equatorial points of their respective spheres. The diffraction pattern produced by HOPS/HyOPS beams can be used to identify the polarization singularity index and the sphere on which the singular beam is represented. But it does not reveal the location of the point on the sphere. All the polarization distributions represented by points on a given latitude of a sphere have identical diffraction intensity distribution^48^. To remove this diffraction degeneracy, further polarization transformations are needed.

**Figure 2:**
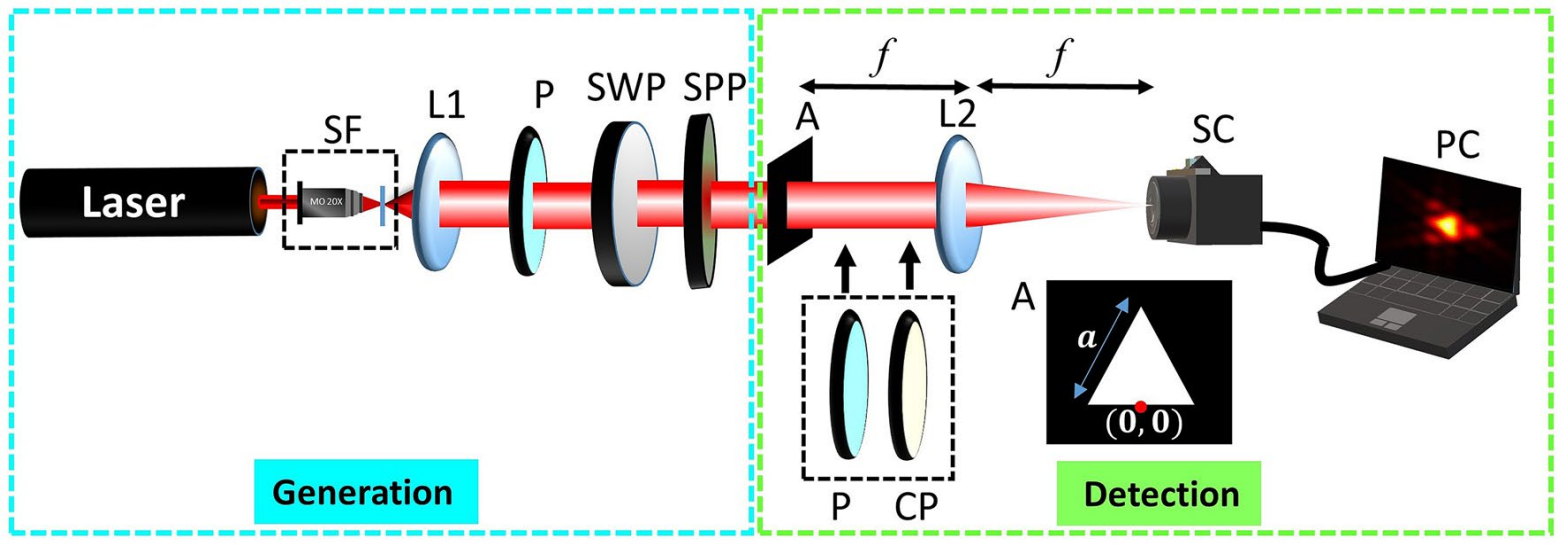
Experimental set up for lifting the degeneracy of Stokes index states: *L1, L2* lenses, *P* polarizer, *SF* spatial filter assembly, *SWP* S wave plate, *SPP* spiral phase plate, *A* triangular aperture, *SC* Stokes camera, *PC* personal computer, *CP* circular polarizer.

The transmittance function of an equilateral triangular aperture with side length *a* can be mathematically written as:

4 $$\begin{aligned} T(x,y) = {\left\{ \begin{array}{ll} 1 &{} \frac{-a}{2}\le x\le \frac{a}{2}\quad \text {and} \quad 0 \le y \le -\sqrt{3}\left( |x|-\frac{a}{2}\right) \\ 0 &{}\text {otherwise} \end{array}\right. } \end{aligned}$$

**Figure 3:**
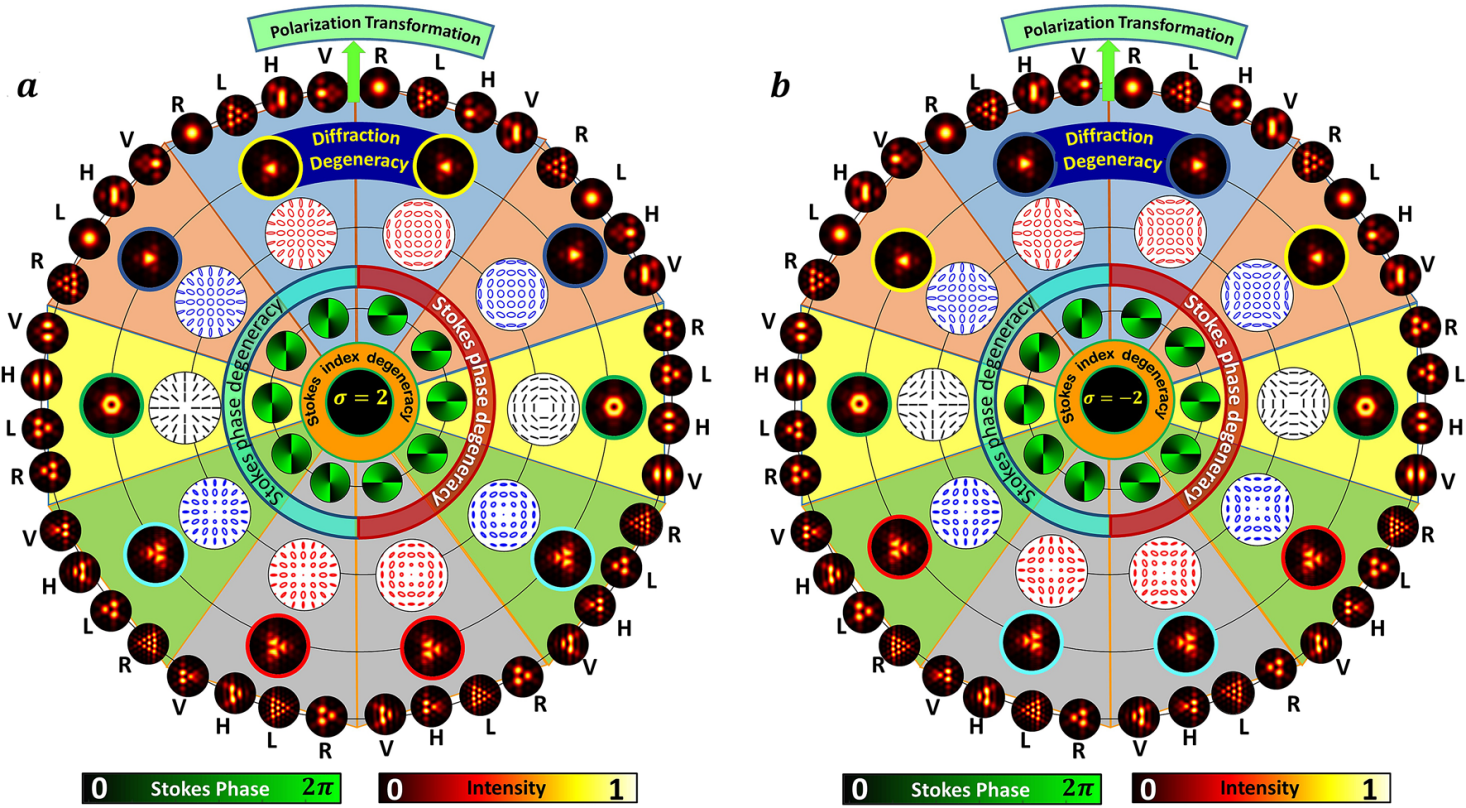
Simulation results depict the degeneracy present in Stokes index (**a**) $\sigma _{12}=2$ and (**b**) $\sigma _{12}=-2$. Stokes phase and Stokes index degeneracies are depicted in the figure. Diffraction degeneracies are shown with same color of borderline. Intensities after polarization transformation corresponding to each polarization distribution are shown in the outer region of the circle. R, L, H and V stand for right circular, left circular, horizontal and vertical polarization components respectively. For positive and negative Stokes indices the HyOPS/HOPS are different.

The field just after the aperture is given by:

5 $$\begin{aligned} {\left| {E_{T}}\right\rangle }=T(x,y){\left| {\psi _{l,m}}\right\rangle } \end{aligned}$$

The Fraunhofer diffraction pattern at the back focal plane of lens with focal length *f* is given by:

6 $$\begin{aligned} {\left| {F}(u,v)\right\rangle }=\frac{A}{i\lambda f} \int _{-\infty }^{\infty } \int _{-\infty }^{\infty } T(x,y){\left| {\psi _{l,m}}\right\rangle } e^{-i\frac{2\pi }{\lambda f}(ux+vy)}dxdy \end{aligned}$$

where (*u*, *v*) are the coordinates at the Fourier plane, *A* is a constant and $\lambda$ is the wavelength of light. The diffraction patterns of orthogonal polarization components are obtained independently. Notably, one may lift the degeneracy present in the Stokes index state by using different apertures as well but the resultant diffraction pattern will depend on the shape and symmetry of the aperture. For instance, using a diamond aperture one cannot differentiate between two oppositely charged vortices of same magnitude ($+l$ and $-l$) due to its horizontal and vertical line symmetry. On the other hand, diffraction patterns of two oppositely charged vortices are rotated by $\pi$ with respect to each other due to the absence of horizontal line symmetry for equilateral triangular aperture. As polarization singularities are the superposition of scalar vortices (with charges *l* and *m*) in orthogonally circularly polarized states, the resultant diffraction intensity of polarization singularities is found to be the sum of diffraction intensities of scalar vortices.

**Figure 4:**
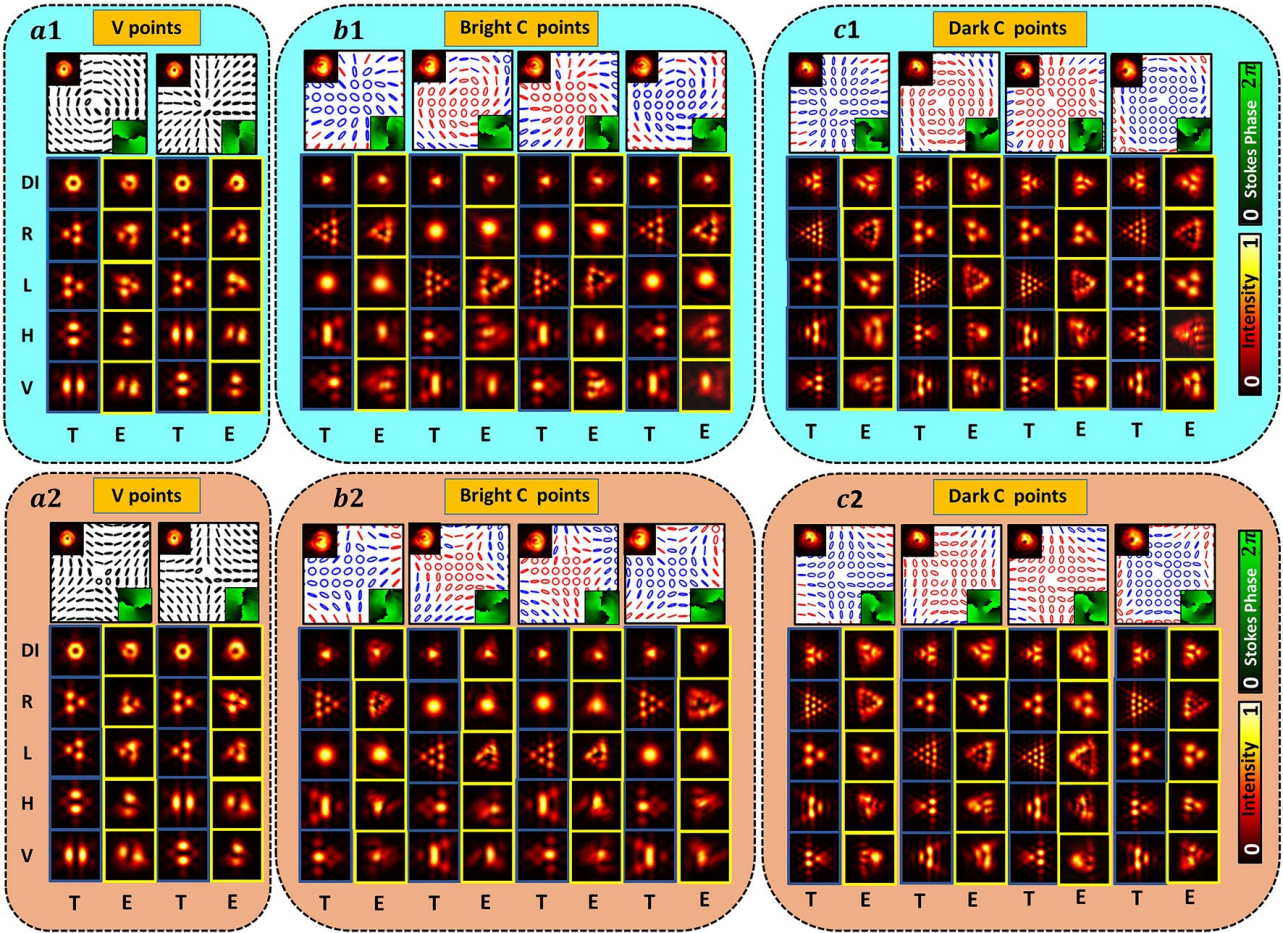
Experimental results depict the input polarization distributions, corresponding diffraction intensities and resultant intensities after polarization transformations for few $\sigma _{12}=2$ (cyan color background) and $\sigma _{12}=-2$ (peach color background) beams. Boxes (**a1**,**a2**) show experimental results for V-points from two different HOPS (one for $\sigma _{12}=2$ and another for $\sigma _{12}=-2$). Similarly, boxes (**b1**,**b2**) and (**c1**,**c2**) show experimental results for bright C-points and dark C-points respectively. Row DI represents resultant diffraction intensity. R, L, H and V represent right circular, left circular, horizontal and vertical polarization component’s intensities respectively. T and E represent columns for theoretical and experimental results respectively.

### Experimental setup

The experimental setup used for detecting degenerate Stokes index states is shown in Fig. 2. A combination of the S wave plate (SWP)^58^ and spiral phase plate (SPP) is used to generate different combinations of topological charges *l* and *m* in orthogonal spin-orbit states. A collimated beam from He Ne laser passing through the SWP produces V-point singularity of one type (radial or azimuthal) depending on the input polarization state (horizontal or vertical). A half-wave plate after SWP can be used to invert the sign of Stokes index^59^. The spiral phase plate after S wave plate converts the V-point into bright or dark C-point depending on the charge of the SPP. For example, a spiral phase plate placed at charge 1 (2) will give a combination of vortices in orthogonal spin-orbit states with charges 0 (3) and 2 (1) and hence produce bright (dark) C-point. A triangular aperture is inserted in the beam path to produce diffraction pattern and polarization transformation is carried out by inserting linear or circular polarizers in the setup. A Stokes camera is used to capture the diffraction intensity patterns and resultant intensities after polarization transformation at the output. For plotting the polarization distribution of the input beams, Stokes parameters are measured using Stokes camera (SALSA, Bossa Nova Technologies, USA). Using the experimentally measured Stokes parameters, the ellipticity and azimuth parameters are calculated at each of the pixel. From these polarization ellipse parameters, the SOP distribution is obtained. The setup used here for generating input beams, can be used to produce polarization singularities with even Stokes index values. The input beams with any arbitrary Stokes index value can be generated using modified Mach-Zehnder type interferometer^57^.

## Results and discussion

In Fig. 3, we discuss various sub degeneracies associated with Stokes index including Stokes phase and diffraction degeneracy by considering two Stokes indices of same magnitude but with different polarity (a) $\sigma _{12}=2$ (left side) and (b) $\sigma _{12}=-2$ (right side). Starting from the center where the Stokes index $\sigma _{12}$ is given, the first inner circle shows few of the degenerate Stokes phase distributions. The background color for each Stokes index figure (in left and right side) indicates the cases belonging to the same sphere. Comparing with Fig. 1, for example yellow background corresponds to HOPS, whereas other four different color backgrounds are for four different HyOPS.

**Figure 5:**
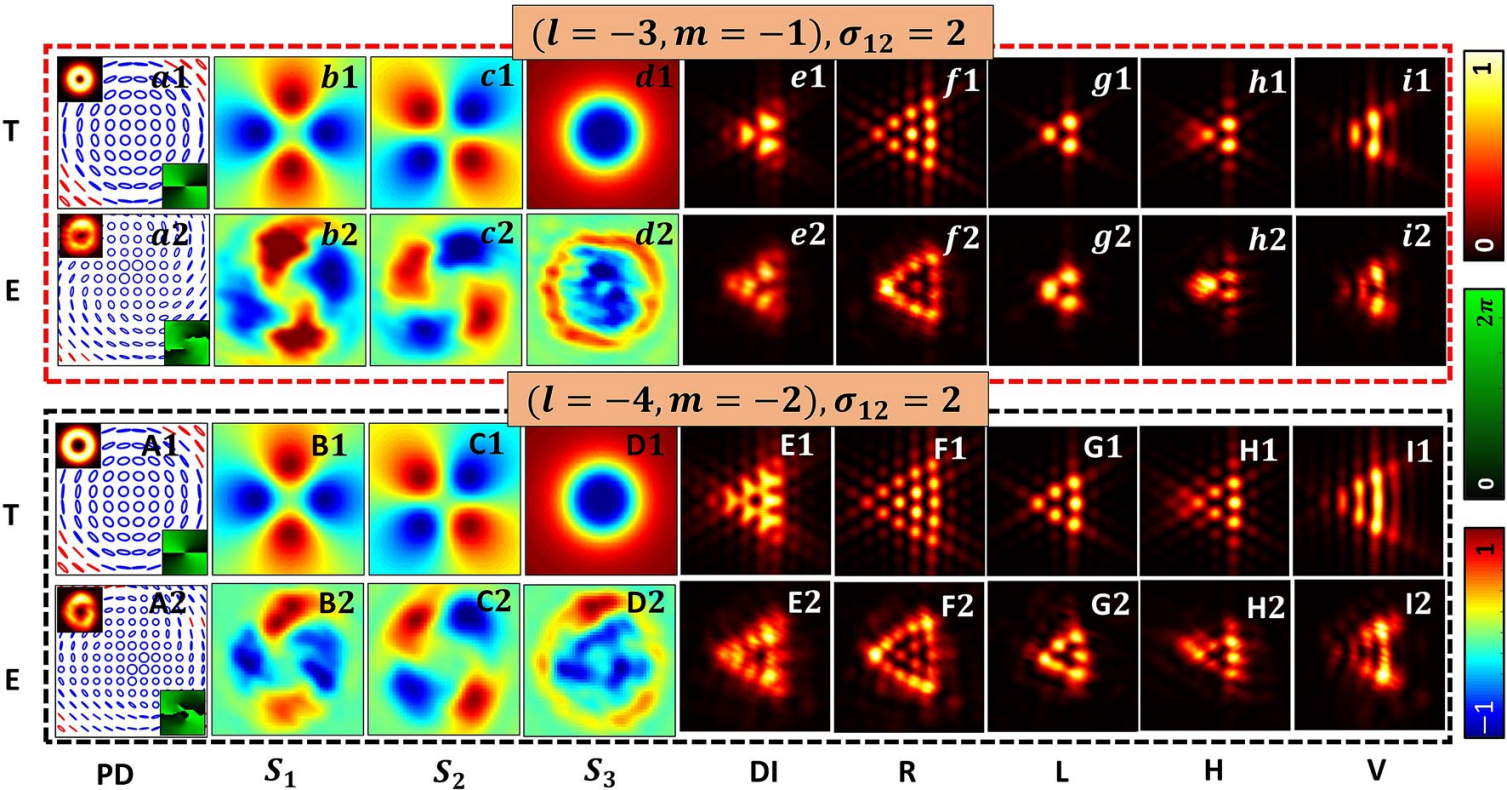
Detection of OAM and SAM content of beams possessing alike polarization distributions. Polarization distributions, Stokes parameters distributions and diffracted intensity patterns for $\sigma _{12}=2 (l=-3, m=-1)$ and $\sigma _{12}=2 (l=-4, m=-2)$ are given in red (top) and black (bottom) boxes respectively. Abbreviations for different columns (a-i, A-I) are, PD: polarization distribution, $S_1, S_2, S_3$ are Stokes parameters distributions, DI: diffraction intensities, (R, L, H, V): diffracted component intensities. Notations T and E are used to represent rows carrying theoretical and experimental results. Note for a dark C point since $S_1=S_2=S_3=0$, the attributes such as SOP, handedness, and azimuth can be determined only by a limiting process from its neighbourhood^36^.

**Figure 6:**
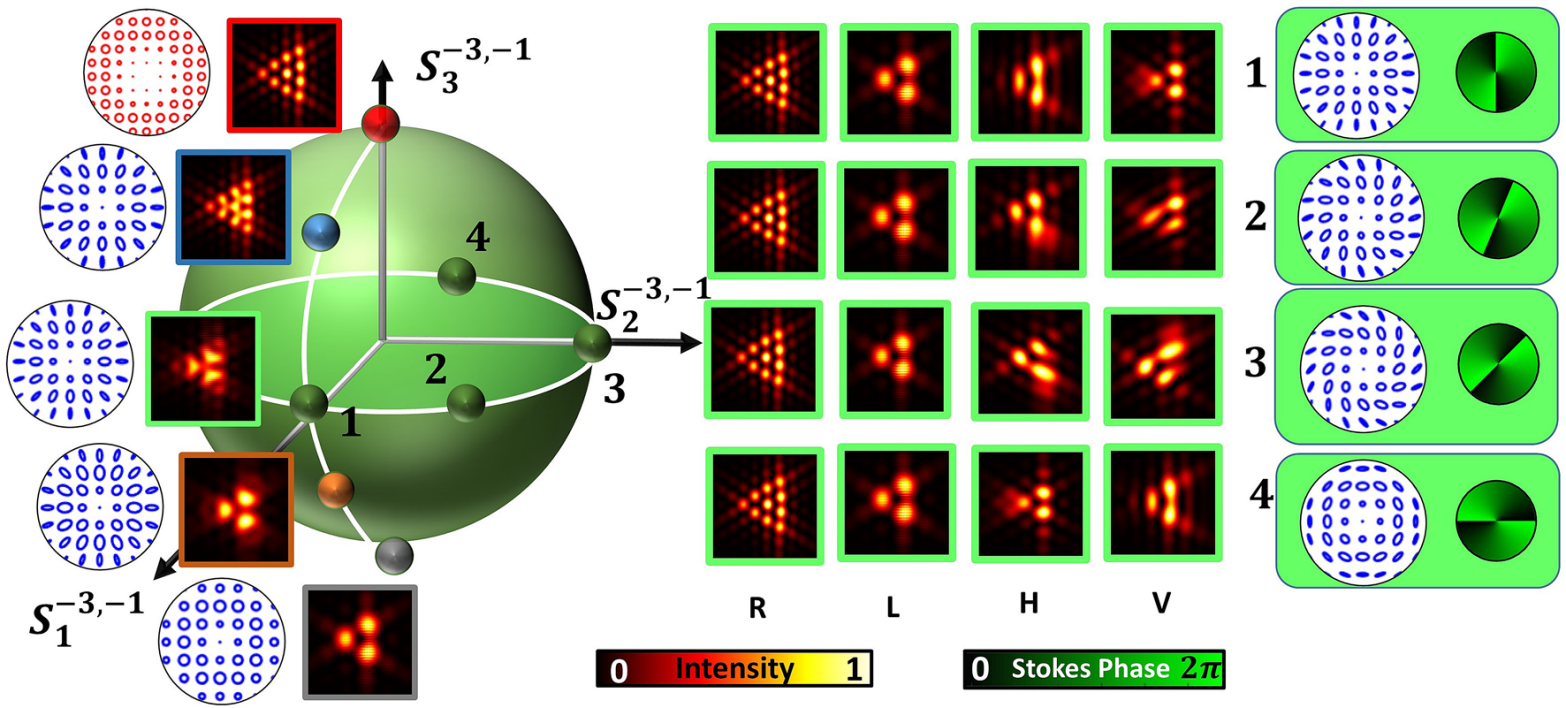
Simulation: Diffraction intensities and polarization distributions of various dark C-points represented by points on a longitude of a HyOPS. For few beams that are diffraction degenerate, represented by equatorial points, marked as 1-4 on the equator, the diffraction patterns are subjected to polarization transformations (R, L, H, V) and are depicted. Their respective SOP distributions and Stokes phases are shown with a green background (extreme right).

Two Stokes phase distributions from each sphere are considered, although an infinite number of Stokes phase distributions share the same Stokes index. Going outward, the SOP distributions and the diffraction patterns are presented. Note that in Fig. 3, the diffraction pattern produced by the beams lying on a specific sphere (along equator) is identical. Hence, diffraction patterns can only reveal as to which sphere the SOP distributions belong. Further discrimination among various SOP distributions can be obtained by polarization transformations, and the patterns obtained are shown in the outermost ring. Polarization transformations segregate the vertical, horizontal, left and right circular polarization component intensities by using linear and circular polarizers - each in two different orientations respectively. As seen in Fig. 3 (outermost ring), the handedness of the C-point is reflected in circular polarization components and the linear polarization components decode the SOP distribution belonging to the same sphere.

Experimental results for beams with index $\sigma _{12}=2$ (cyan color background) and $\sigma _{12}=-2$ (peach color background) are shown in Fig. 4. The first row in these figures shows the experimentally obtained input beams’ polarization distribution with insets representing corresponding intensities and Stokes phases. Rows DI; R, L, H and V in figures represent total diffraction intensity; right circular, left circular, horizontal and vertical polarization components’ intensities respectively. Columns ‘T’ and ‘E’ represent theory and experiment. Note that many C-points/ V-points with the same Stokes index can be obtained by various combinations of *l* and *m* as long as $m-l=\sigma _{12}$ remains constant (refer Fig. 1). Therefore each of these C-points/V points belong to different HyOPS/HOPS. In Fig. 4, segregation of different SAM and OAM states using diffraction and polarization transformation is shown for beams with identical Stokes index/ Stokes phase but with different polarization distributions. By noting the different combinations of diffraction and polarization patterns, Stokes degenerate states can be distinguished. But in case of dark C-points and points on the same longitude of a HyOPS, one can observe polarization distribution degenerate states as well. Stokes polarimetry is used to find the polarization distributions of input beams in terms of intensity measurements in different orthogonal basis states. Due to identical polarization distribution of these HyOPS beams, Stokes polarimetry also fails to distinguish between them. These two cases of polarization distribution degeneracy are shown in Figs. 5 and 6.

Dark C-points of same Stokes index, same Stokes phase, same handedness and identical polarization distribution can be obtained using many combinations of optical vortices such that $\sigma _{12}=m-l$. In Fig. 5, it can be seen that Stokes polarimetry correctly gives the polarization distribution for two such dark C-points that have same Stokes index. But, these two C-points belong to different HyOPS and Stokes polarimetry fails to distinguish this aspect. In Fig. 5, red (top) and black (bottom) show simulation (T) and experimental (E) results for two dark C-points with Stokes index $\sigma _{12}=2 (l=-3, m=-1)$ and $\sigma _{12}=2 (l=-4, m=-2)$ respectively. Here, one can clearly see how our method provides correct information which Stokes polarimetry fails to provide. Although V-points^20–24^ and higher index C-points^57,60^ can be experimentally generated, they are not stable and tend to disintegrate under strong perturbation. In the experimental figures we observed that the $S_3$ values are very low around V-points indicating that there is no split. The split seen in the Stokes phases may be due to experimental artifact. In the experimental set-up used, the input beams are generated using the combination of SWP and SPP. Hence, the pair of vortices are not generated at the same plane. Due to the different propagation distances encountered by the two superposing beams, the resulting intensities in the experimental results are not exactly matching with the theoretically predicted results. Further, it is difficult to ensure precise core to core alignment of the V-point beam and the vortex beam generated by SWP and SPP respectively in the experiment.

Figure 6 depicts many intensity patterns for various HyOPS beams diffracting through a triangular aperture. Five beams represented by five longitudinal points and four beams represented by four equatorial points as depicted on the HyOPS of Fig. 6 were selected for this study. The input HyOPS beams’ SOP distribution and diffracted intensity patterns are depicted near the points along the longitude on the sphere, whereas for the equatorial points the diffraction patterns are further subjected to polarization transformations and are shown on the right side of Fig. 6 in a 4x4 frame along with their SOP distributions and Stokes phases. Beams falling on a different latitude (other than equator) possess different relative weightage of the superposing OAM states but alike polarization distribution. This feature is also not discriminated when one uses Stokes polarimetry but can easily be detected using diffraction. For the beam represented by a polar point diffraction pattern corresponding to one of OAM states in the superposition alone is present. In the other Pole the pattern corresponding to the other OAM state in the superposition can be seen. Therefore for the diffracted beams represented by equatorial points use of circular polarizer reveals OAM content in these two eigen-polarization states. All the beams represented by points on a particular latitude produce same diffraction patterns (for equatorial points it is shown in Fig. 6), and by the patterns obtained in polarization transformation, lifting of diffraction degeneracy can be witnessed.

## Conclusion

In this work, we have identified the various possible Stokes index degenerate states in the context of inhomogeneous HOPS/HyOPS beams. The other form of subset degeneracies occurring in Stokes phase, diffraction pattern, and polarization distribution associated with a fixed Stokes index are also discussed. We have shown that Stokes index state degeneracy can be lifted by a method based on combination of diffraction and polarization transformation in linear and circular basis. A triangular aperture is used to diffract these spin-orbit beams and it is realized that the overall diffracted intensity pattern can be used to decide to which sphere, (HyOPS or HOPS) the Stokes index is attributed. Further, polarization transformation that segregates the component intensities in the diffraction pattern, pin-points the location on the sphere that represents the SOP distribution. Distinction among beams endowed with V-points, bright C-points, dark C-points singularity and their handedness is demonstrated through simulation and supported by experimental results. Apart from being simple and elegant intensity based measurement, our method successfully distinguishes polarization degenerate HyOPS beams where even Stokes polarimetry fails. This study provides a systematic guide for intensity based detection techniques for the unambiguous detection of spin-orbit beams.
